# Application of biosurfactant from *Bacillus subtilis* C9 for controlling cladoceran grazers in algal cultivation systems

**DOI:** 10.1038/s41598-018-23535-8

**Published:** 2018-03-29

**Authors:** Jin-Ho Yun, Dae-Hyun Cho, Bongsoo Lee, Hee-Sik Kim, Yong Keun Chang

**Affiliations:** 1 Department of Chemical and Biomolecular Engineering, KAIST, 291 Daehak-ro, Yuseong-gu, Daejeon 305-701 Republic of Korea; 2 Cell Factory Research Center, Korea Research Institute of Bioscience and Biotechnology (KRIBB), Yuseong-gu, Daejeon 305-806 Republic of Korea; 3 Green Chemistry and Environmental Biotechnology, University of Science and Technology (UST), Yuseong-gu, Daejeon 305-350 Republic of Korea; 4 Advanced Biomass R&D Center, 291 Daehak-ro, Yuseong-gu, Daejeon 305-701 Republic of Korea

## Abstract

Open algal cultivation platforms often suffer crop losses to herbivorous grazers that have potential to devastate biomass production within a few days. While a number of studies suggest synthetic chemicals as control agents for voracious algal grazers, environmental and safety concerns associated with the use of these chemicals encourage the exploration of alternative biological control agents. We hereby propose the application of a biosurfactant produced by *Bacillus subtilis* C9 (referred to as C9-biosurfactant) for controlling cladoceran grazers commonly found in algal cultivation systems. The results indicated that C9-biosurfactant completely eradicated *Daphnia pulex* and *Moina macrocopa* within 24 hours when concentrations were equal to or exceeded 6 mg/L. Moreover, supplying C9-biosurfactant into the cultures of selected algal species with and without cladoceran grazers indicated no adverse effect of C9-biosurfactant on the growth and lipid productivity of algal crops, while cladocerans were selectively controlled by C9-biosurfactant even under the presence of their prey. These results thus indicate that C9-biosurfactant could be an effective biocontrol agent for cladoceran grazers at industrial algal cultivation.

## Introduction

Anthropogenic climate change and population growth have resulted in a global-scale consensus to mitigate the usage of unsustainable energy sources, and concomitantly initiated active research and investment efforts to at least partially replace fossil fuels with renewable and carbon-neutral energy sources^1^. Microalgae, in particular, have been given considerable attention because of their relatively high growth rates compared to terrestrial biofuel crops and the possibilities of utilizing flue gases and excess nutrients in wastewater sources for their prolific growth^2,3^. Nonetheless, the production of microalgae-derived biofuels and bioproducts is still at the explorative stage, and a number of challenges have arisen from mass cultivation of algal biomass to downstream processes for producing target products^2–6^. Especially, achieving high and reliable biomass production at the industrial-scale poses a great and pressing challenge to make entire production chain cost-competitive^7,8^.

While the mass production of algal biomass is primarily achieved in outdoor non-sterile systems due to the constraints associated with the vast capital and operational costs of sterile closed systems, the foremost issues in outdoor cultivation of short-listed algal species for the production of biofuels and bioproducts are culture contamination and population crashes^9–11^. In particular, the grazing pressure of herbivorous zooplanktons can cause significant reductions in productivity and quality of harvested biomass, which in turn cause losses in overall profitability^12^. Cauchie *et al*., for example, measured a 99% reduction in algal chlorophyll-a due to *Daphnia* grazing over several days in an open pond system^12,13^. Similarly, a one-year-long operation of wastewater-fed open algal ponds experienced repeated outbreaks of cladoceran *Daphnia*, and it was subsequently confirmed in laboratory that this cladoceran grazer imposed 12.5–87.87% reductions in the dry cell weight (DCW) of common microalgal strains found in outdoor open ponds^14^. In addition, White and Ryan estimated algal crop losses to grazers at 20% ± 10% based on their industrial-scale operation of outdoor algal ponds^15^. It is therefore imperative to develop cost-effective strategies for controlling unpredictable blooms of algal grazers because loss of even a single algal reactor in an array can dramatically impact overall facility productivity and yield^9,11,15,16^.

Synthetic chemicals have thus been explored to mitigate algal crop losses from zooplankton grazers in algal production systems^17–21^. For example, the disinfectant hypochlorite was used to control the rotifer *Brachionus calyciflorus* in laboratory cultures of the microalga *Chlorella kessleri*^19,21,22^. In addition, Wang *et al*. outlined the lethal concentrations of three synthetic pesticides on *B. calyciflorus* and *Daphnia pulex*^23^. Synthetic compounds, however, may have negative impacts on non-target organisms and/or adjacent ecosystems^24,25^. Therefore, sustainable cost-effective strategies that control growth and proliferation of grazing zooplanktons in algal cultivation platforms without the use of synthetic chemicals are needed^17,26–30^.

In terrestrial agriculture, microbial secondary metabolites have been regarded as promissory alternatives to synthetic pesticides because of their diverse metabolites with high biological activities^31,32^. In particular, *Bacillus* spp. lead active research and development efforts because the strains of *Bacillus* are known to excrete peptides and lipopeptides, such as fungicine, iturin, bacillomicine, and others that have antifungal, antibacterial, and high surfactant activity^33^. Although a variety of biological control products based on *Bacillus* species are available for agronomical use to control agricultural pests, few studies explored the direct application of the secondary metabolites of *Bacillus* in the systems targeting to grow algal biomass^33^.

Previously, Kim *et al*. isolated *Bacillus subtilis* C9 that produces surfactin, a lipopeptide biosurfactant with antibacterial, antiviral, and anti-biofilm forming activities^34,35^. While *B. subtilis* and its biosurfactant were known to exhibit biocontrol activities against mosquito pupae and phytopathogenic fungi^31,36–38^, the purpose of this study was to explore the biocontrol activity of recovered biosurfactant from the culture of *B. subtilis* C9 (referred hereupon as C9-biosurfactant) on common cladoceran grazers in algal cultivation platforms^14,39^. Grazer-introduced cultures were first subjected to different concentrations of C9-biosurfactant to test whether cladoceran grazers were effectively controlled by C9-biosurfactant. Because the industrial applicability of any biocontrol compound must assure its non-toxicity to non-target species, we further explored the influence of supplemental C9-biosurfactant on the growth and/or lipid productivity of two microalgal strains with and without the presence of cladoceran grazers.

## Results and Discussion

### Recovery and identification of C9-biosurfactant

The average surface tension of cell-free supernatants from triplicate cultures of *B. subtilis* C9 after a 72-hour-long cultivation period was 29.8 ± 1.1 mN/m, and the average concentration of acid precipitate in the respective culture supernatant was 0.9 ± 0.2 g/L. 0.3 ± 0.1 g/L of C9-biosurfactant was then recovered from acid precipitates by dissolving C9-biosurfactant in demineralized water and freeze-drying the biosurfactant solution. Our subsequent mass spectrometry analysis of C9-biosurfactant exhibited the highest peak at *m/z* 1058.67, where the highest peak of HPLC-grade surfactin from *B. subtilis* was also observed (Fig. S1). While the molecular ion species of surfactin can be detected in either protonated or sodium adduct forms, Kim *et al*. also reported a high peak of C9-biosurfactant at *m/z* 1058, where the sodium adduct of surfactin is putatively present^34,35,40^. In addition, the CMD values of aqueous solutions of C9-biosurfactant and HPLC-grade surfactin at an identical concentration of 0.3 g/L were 50 and 60, indicating that the relative proportion of surfactin in C9-biosurfactant was roughly 83% (Figs S2 and S3). These results thus indicated the production of biosurfactant in the culture of *B. subtilis* C9 and further confirmed surfactin as the main surfactant compound of C9-biosurfactant^34,35,40^. Although C9-biosurfactant tested in this study was obtained from triplicated batch cultivation of *B. subtilis* C9, it should be also noted that the concentration of surfactin in the culture of *B. subtilis* C9 could substantially vary under different cultivation conditions, and direct quantification of surfactin in C9-biosurfactant will be required especially when determining the application doses of C9-biosurfactant in industrial algal cultivation^41^.

### C9-biosurfactant acts as an effective control agent for cladoceran Daphnia and Moina

**Figure 1:**
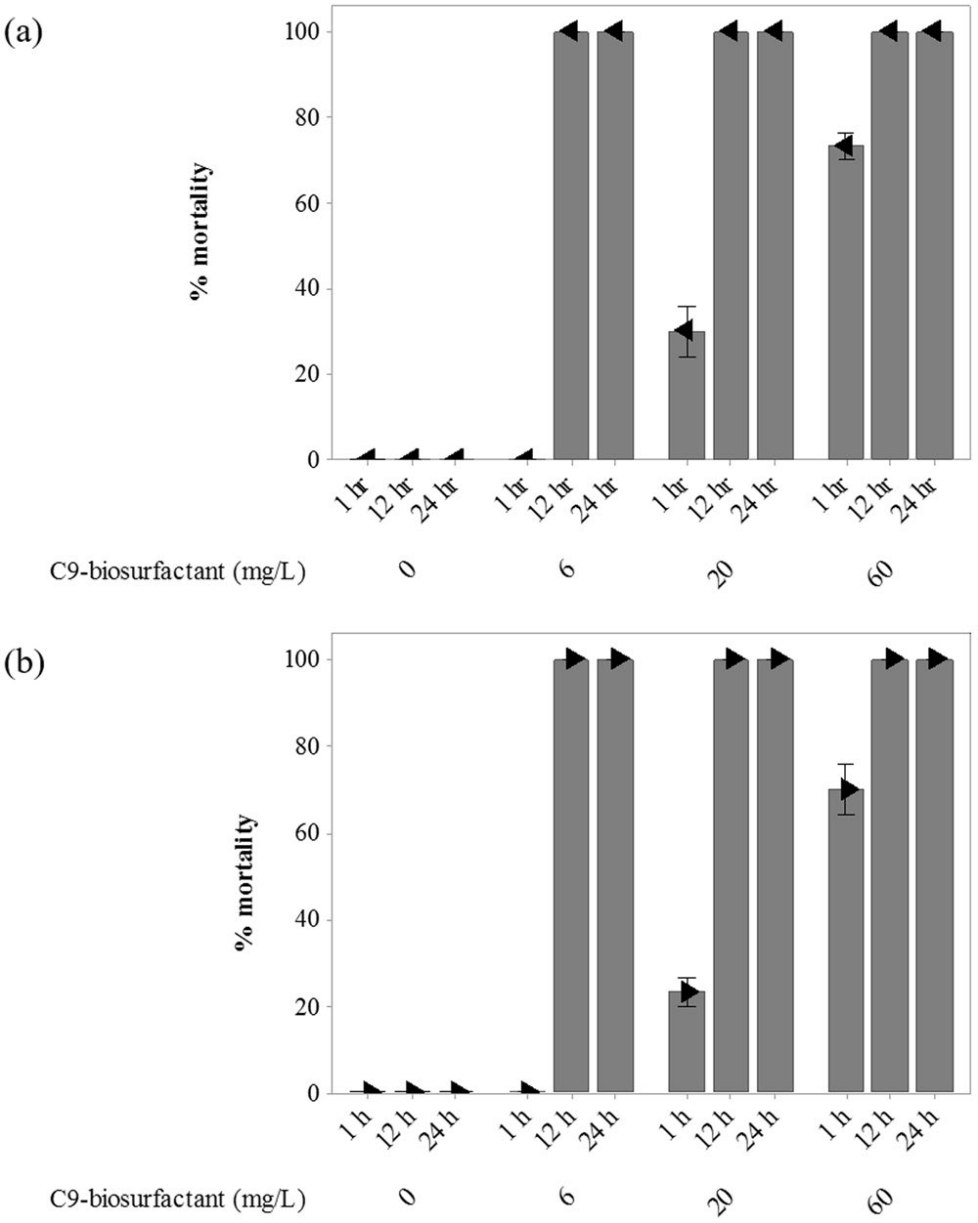
The % mortalities of *Daphnia pulex* (**a**) and *Moina macrocopa* (**b**) after 1, 12, and 24 hr of exposure to C9-biosurfactant. Individuals settled on the bottom of 10-mL glass tubes without visual movement after gentle shaking were counted as dead. Error bars denote standard error of the mean.

While a diverse array of physical, biological, and chemical measures were recently explored to successfully control blooms of algal grazers^22^, our results indicated that C9-biosurfactant can also be implemented to eradicate cladoceran grazers in industrial algal cultivation platforms. The results showed 6 mg/L of C9-biosurfactant was enough to cause complete mortality of both *Daphnia pulex* and *Moina macrocopa* within 12 hours (Fig. 1). Our subsequent test with HPLC-grade surfactin from *Bacillus subtilis* also indicated the complete mortality of cladocerans within 12 hours at concentrations equal to or above 6 mg/L, confirming that the main surfactant compound of C9-biosurfactant resulted in the mortality of algal grazers (Figs S2 and S5).

The results further suggested that the cladoceran-control activity of C9-biosurfactant is dependent on the concentration of C9-biosurfactant. In particular, the data obtained 1 hour after the application of C9-biosurfactant exhibited substantial increases in the percent mortalities of both cladocerans with increasing concentrations of C9-biosurfactant (Fig. 1). While Vollenbroich *et al*. similarly reported that the antiviral activity of surfactin substantially increased with increasing surfactin concentration^42^, dose-dependent mortality response of cladocerans may provide important information that can be exploited to achieve successful cladoceran-control in a shorter period of time. Nonetheless, complete eradication of cladoceran grazers within 1 day following the application of C9-biosurfactant at all tested concentrations indicates C9-biosurfactant can act as an effective biocontrol agent for cladoceran algal grazers in industrial algal cultivation systems, especially because invading cladoceran grazers often require at least 1–2 days to substantially consume dominant microalgal species^22,39,43^.

Although this study was focused on demonstrating the biocontrol activity of C9-biosurfactant on cladoceran grazers, microzooplanktons, including rotifers and ciliated protists, are also known to detrimentally contaminate industrial algal cultivation platforms. Indeed, order-of-magnitude reductions in microalgal biomass have been well acknowledged for ciliate-dominated zooplankton in commercial *Spirulina* (*Arthrospira*) cultivation sites and for rotifer-dominated zooplankton in High Rate Algal Ponds (HRAPs)^22,29,39,43,44^. Such devastatingly high level of algal crop losses will impede making algae-based products a commercial reality, thus exploring the effectiveness of C9-biosurfactant in controlling other frequent algal grazers will be vitally important to further support the implementation of C9-biosurfactant at industrial algal cultivation.

Indeed, previous studies have reported that surfactants generally exhibit biocontrol activities against different organisms^38,45,46^. Geetha *et al*., for example, noted the mosquito pupicidal activity exhibited by the biosurfactant of *Bacillus subtilis*, and Manonmani *et al*. further confirmed that the culture supernatant of *Bacillus subtilis* was found to kill both larval and pupal stage of mosquito species of *Anopheles stephensi*, *Culex quinquefasciatus*, and *Aedes aegyti*, while a significantly lesser amount of biosurfactant was required to kill the mosquito pupae^38,45^. In addition, Lechuga *et al*. explored the toxicity of different surfactants on the luminescent bacterium *Vibrio fischeri*, and reported that *V. fischeri* was more sensitive to the toxic effects of the surfactants than was cladoceran *Daphnia*^46^. Nonetheless, it is evident that the cladoceran-control activity of C9-biosurfactant bears substantial industrial potential for managing blooms of cladoceran grazers in algal cultivation platforms^29,47^, especially given that large-bodied cladocerans are typically thought to control microalgal production much more effectively than other small-bodied herbivorous grazers because their algal ingestion is less influenced by the size of resident algal strains^29,47^.

Although the exact mode of action of allelopathic C9-biosurfactant on cladoceran grazers requires further exploration, it was speculated that a reduction in the surface tension of the water caused by C9-biosurfactant was likely to require more energetic costs during swimming that may have substantially contributed to the observed cladoceran mortality^36,37^. Vollenbroich *et al*. reported the disintegration of mycoplasma membranes after treating mycoplasma-contaminated mammalian cells with surfactin, and concluded that the disintegration of bacterial membrane was due to a physicochemical interaction of the membrane-active surfactant with the outer part of the lipid membrane bilayer, whereas the low cytotoxicity of surfactin for mammalian cells was observed^42,48^. Although the cladoceran-control activity of C9-biosurfactant is likely to depend on the life cycle and nutritional status of cladocerans^49,50^, a physicochemical interaction of surfactin with the carapace of cladocerans may also be an important contributor to the observed cladoceran mortality^36,37,42,48,51^.

### C9-biosurfactant does not negatively influence the biomass and lipid productivity of algal crops

**Figure 2:**
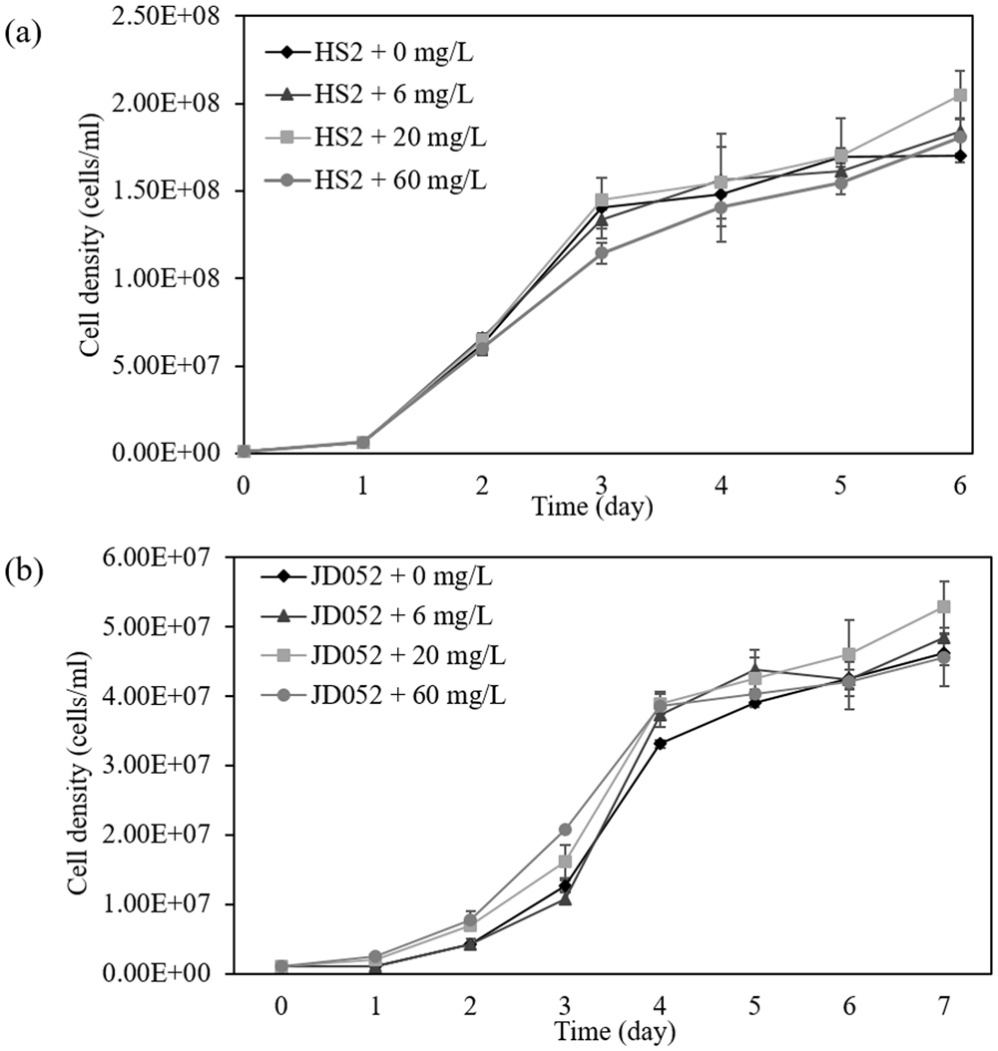
The growth curves of *Chlorella* sp. HS2 (**a**) and *Scenedesmus deserticola* JD052 (**b**) throughout the batch cultivation in 250-mL flasks under varying concentrations of C9-biosurfactant in BG-11 medium. Error bars denote the standard deviation from the mean of triplicate independent measurements.

Any potential biocontrol agents in agriculture should be tested for their toxicity to crop species prior to warranting their application in agricultural fields^33^. Likewise, the application of C9-biosurfactant in algal cultivation systems cannot be justified without evaluating the influence of C9-biosurfactant on the growth and productivity of selected algal crops. The results of our flasks-scale experiments, however, clearly indicated no growth inhibiting effect of C9-biosurfactant on *Chlorella* sp. HS2 and *Scenedesmus deserticola* JD052 (Fig. 2 and Table 1).

**Table 1:** Specific growth rates of *Chlorella* sp.

	0 mg/L of C9-biosurfactant	6 mg/L of C9-biosurfactant	20 mg/L of C9-biosurfactant	60 mg/L of C9-biosurfactant
*Chlorella* sp. HS2	1.55 ± 0.21	1.52 ± 0.28	1.56 ± 0.42	1.20 ± 0.11
*Scenedesmus deserticola* JD052	1.1 ± 0.21	1.18 ± 0.34	1.08 ± 0.27	0.929 ± 0.18

HS2 and *Scenedesmus deserticola* JD052 under varying concentrations of C9-biosurfactant in 250-mL flask cultures. Data are presented as means ±95% confidence intervals.

The specific growth rates of each algal species were not statistically different under varying concentrations of C9-biosurfactant (p-values > 0.05), except the culture of *Chlorella* sp. HS2 amended with 60 mg/L of C9-biosurfactant, which had a lower specific growth rate than that of the control without C9-biosurfactant (p-value < 0.05) (Table 1). The well-plate experiments similarly indicated that the supplemental surfactin did not negatively influence the growth of *Chlorella* sp. HS2 and *Scenedesmus deserticola* JD052, except in the case of adding 60 mg/L of surfactin into the culture of *Chlorella* sp. HS2 where the final cell density was only about half that of the control with no supplemental surfactin (Fig. S7). These results indicated no growth inhibiting effect of C9-biosurfactant and surfactin on the selected *Chlorella* and *Scenedesmus* strains at the concentrations below 60 mg/L, which were enough to cause complete cladoceran mortality (Fig. 1).

**Figure 3:**
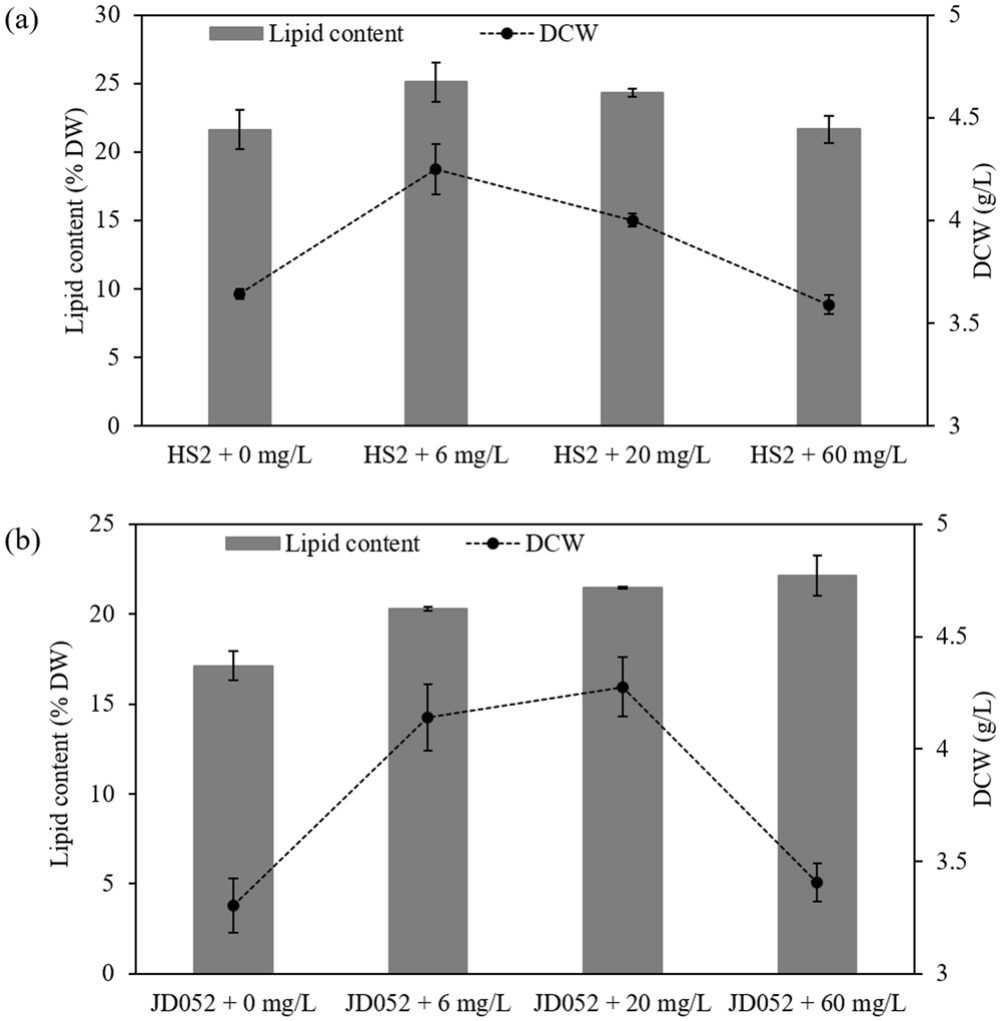
Dry cell weight (g/L) and lipid content (% DW) of harvested biomass of *Chlorella* sp. HS2 (**a**) and *Scenedesmus deserticola* JD052 (**b**) following the harvest of 250-mL flask cultures grown under varying concentrations of C9-biosurfactant in batch cultivation mode. Error bars represent the standard deviation from the mean for triplicate independent measurements.

The DCW and lipid content of *Chlorella* sp. HS2 and *Scenedesmus deserticola* JD052 substantially increased under 6 and 20 mg/L treatments of C9-biosurfactant (Fig. 3). While no substantial increase in final DCW was measured under 60 mg/L of C9-biosurfactant for either algal strain, increasing the concentration of C9-biosurfactant up to 60 mg/L continuously enhanced the lipid content of *Scenedesmus deserticola* JD052 (Fig. 3). In contrast, the lipid content of *Chlorella* sp. HS2 was highest in the cultures amended with 6 mg/L of C9-biosurfactant, and the *Chlorella* cultures amended with 60 mg/L of C9-biosurfactant exhibited no considerable difference in the lipid content compared to that of the control with no supplemental C9-biosurfactant (Fig. 3). The subsequent FAME analysis further indicated minor changes (i.e., 1–5%) in the proportion of each type of fatty acid under varying concentrations of C9-biosurfactant: oleic (C18:1n9c) and α-linolenic (C18:3n3) acids exhibited substantial shifts in the cultures of *Scenedesmus deserticola* JD052 and the additive C9-biosurfactant substantially influenced the proportions of palmitic (C16:0) and linoleic (C18:2n6c) acids in the cultures of *Chlorella* sp. HS2 (Tables 2 and 3).

**Table 2:** Fatty acid composition of *Chlorella* sp.

Fatty acid	Proportion (%) of fatty acid			
	0 mg/L of C9-biosurfactant	6 mg/L of C9-biosurfactant	20 mg/L of C9-biosurfactant	60 mg/L of C9-biosurfactant
Palmitate (C16:0)	29.66 ± 1.34	26.79 ± 1.71	26.44 ± 0.80	25.18 ± 0.37
Palmitoleate (C16:1)	0.30 ± 0.03	0.00 ± 0.00	0.28 ± 0.40	0.65 ± 0.91
Hexadecatrienoate (C16:3)	5.46 ± 0.54	6.55 ± 0.72	7.66 ± 1.72	7.29 ± 0.36
Stearate (C18:0)	1.80 ± 0.21	2.15 ± 0.19	1.56 ± 0.40	1.61 ± 0.15
Oleate (C18:1n9c)	24.72 ± 0.91	23.51 ± 1.62	21.55 ± 2.63	21.46 ± 0.85
Linoleate (C18:2n6c)	27.00 ± 1.16	30.96 ± 1.70	30.72 ± 1.36	32.29 ± 0.31
Linolenate (C18:3n3c)	3.34 ± 0.23	3.67 ± 0.49	4.18 ± 0.78	4.11 ± 0.26
Saturated (%)	31.46 ± 1.55	28.94 ± 1.90	28.00 ± 1.20	26.79 ± 0.30
Monounsaturated (%)	25.02 ± 0.48	23.51 ± 1.62	21.83 ± 3.03	22.10 ± 1.76
Polyunsaturated (%)	35.80 ± 1.93	41.18 ± 2.90	42.57 ± 3.86	43.68 ± 0.93
Others (%)	7.71 ± 0.10	6.37 ± 0.62	7.60 ± 0.37	7.42 ± 0.62
Total (%)	100	100	100	100

HS2 under varying concentrations of C9-biosurfactant. Data are presented as means with the standard deviation.

**Table 3:** Fatty acid composition of *Scenedesmus deserticola* JD052 under varying concentrations of C9-biosurfactant.

Fatty acid	Proportion (%) of fatty acid			
	0 mg/L of C9-biosurfactant	6 mg/L of C9-biosurfactant	20 mg/L of C9-biosurfactant	60 mg/L of C9-biosurfactant
Palmitate (C16:0)	21.92 ± 0.38	20.70 ± 0.14	20.13 ± 0.14	19.30 ± 0.26
Palmitoleate (C16:1)	4.00 ± 0.11	4.41 ± 0.05	3.49 ± 0.40	3.07 ± 0.44
Hexadecatrienoate (C16:3)	5.31 ± 0.12	5.51 ± 0.18	5.64 ± 0.17	5.44 ± 0.26
Stearate (C18:0)	4.67 ± 0.18	3.30 ± 0.11	4.15 ± 0.55	3.90 ± 0.18
Oleate (C18:1n9c)	21.79 ± 0.34	20.58 ± 0.23	20.34 ± 1.42	18.51 ± 0.57
Linoleate (C18:2n6c)	14.46 ± 0.28	15.09 ± 0.08	16.25 ± 0.67	16.72 ± 0.77
Linolenate (C18:3n3c)	13.55 ± 0.39	15.29 ± 0.60	15.07 ± 0.66	17.24 ± 0.13
Saturated (%)	26.59 ± 0.56	24.00 ± 0.25	24.28 ± 0.41	23.20 ± 0.08
Monounsaturated (%)	25.79 ± 0.45	24.99 ± 0.28	23.83 ± 1.83	21.58 ± 1.01
Polyunsaturated (%)	33.32 ± 0.79	35.89 ± 0.35	36.96 ± 1.16	39.41 ± 0.39
Others (%)	14.29 ± 1.79	15.12 ± 0.32	14.92 ± 0.26	15.80 ± 0.54
Total (%)	100	100	100	100

Data are presented as means with the standard deviation.

**Figure 4:**
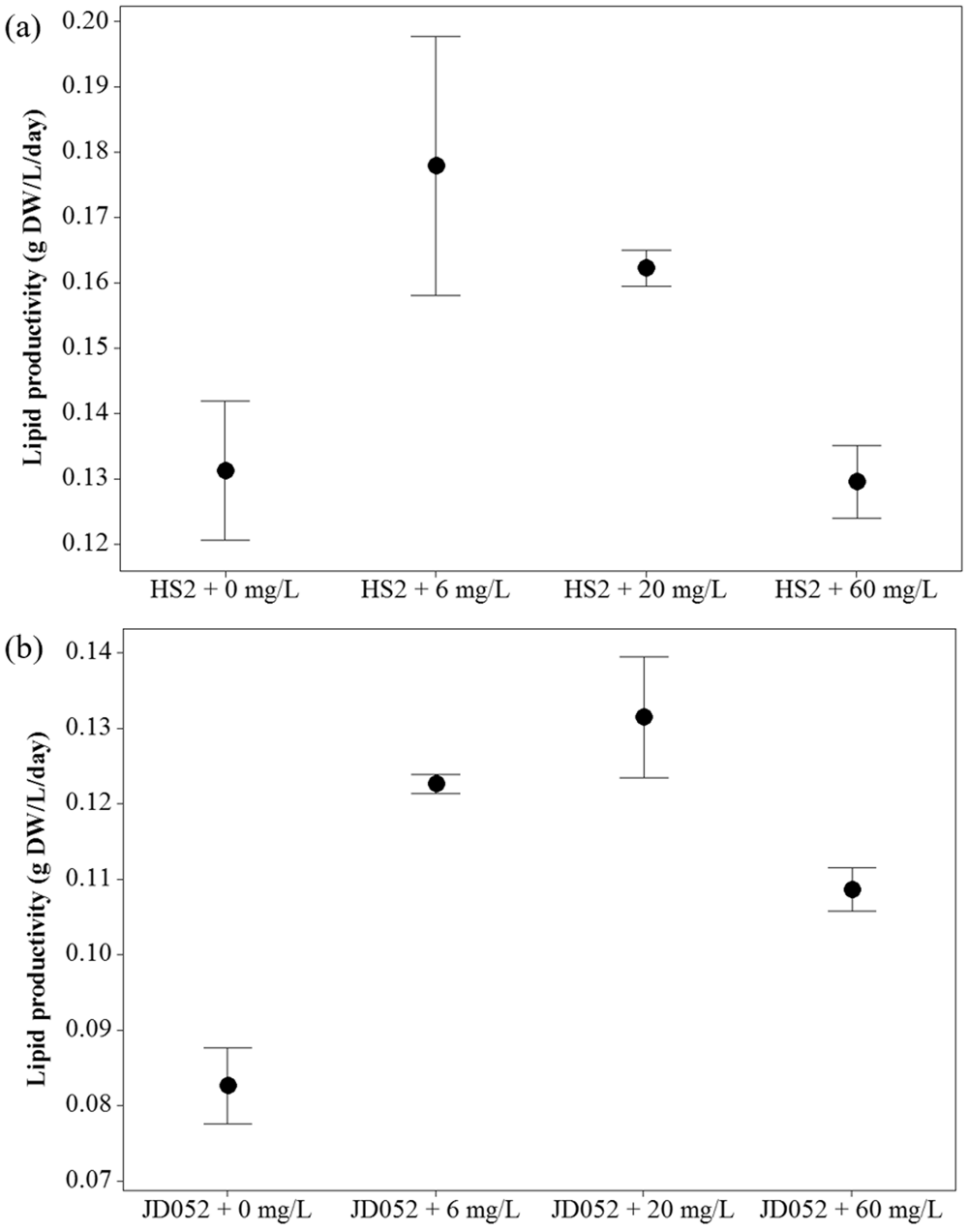
Lipid productivity of the cultures of *Chlorella* sp. HS2 (**a**) and *Scenedesmus deserticola* JD052 (**b**). Lipid productivity was calculated by multiplying lipid content (%) with corresponding biomass productivity after harvesting flask cultures treated with varying concentrations of C9-biosurfactant at the end of either 6 or 7 day-long batch cultivation period. Error bars indicate 95% confidence intervals for the mean.

The lipid productivities of *Chlorella* and *Scenedesmus* were significantly enhanced with the supplemental C9-biosurfactant (p-values < 0.05) in most cases (but see *Chlorella* cultures amended with 60 mg/L of C9-biosurfactant – Fig. 4). The highest lipid productivities of *Chlorella* sp. HS2 and *Scenedesmus deserticola* JD052 were 0.178 and 0.128 g/L/day at C9-biosurfactant concentrations of 6 and 20 mg/L, respectively. Such increments corresponded to 35 and 51% of increases in the lipid productivities compared to the control cultures of each algal strain. While the amount of triacylglycerols (TAG) in harvested algal biomass would be a direct indicator for understanding the influence of supplemental biosurfactant on the bioenergy potential of selected algal strains, Griffiths and Harrison emphasized that lipid productivity itself could be a key characteristic for choosing algal species for biodiesel production^52^. These results thus suggest that the additive C9-biosurfactant does not compromise the growth or lipid productivity of the selected algal crops, and further indicate the possibility of utilizing C9-biosurfactant to promote the bioenergy potential of harvested biomass at industrial algal cultivation.

The increased lipid productivities of *Chlorella* sp. HS2 and *Scenedesmus deserticola* JD052 may be resulted from increased nutrient uptake into cell bodies with the additive C9-biosurfactant. Taoka *et al*., for example, reported ca. 4% increase in the total lipid content of *Thraustochytrium aureum* with the addition of 1% Tween 80, a non-ionic surfactant, and speculated that the addition of surfactant likely interfered with the permeability of cell membranes and enhanced the nutritional uptakes into the cell body^53^. The authors reported significant increases in the proportions of both saturated and monounsaturated fatty acids, while the percentage of unsaturated fatty acids decreased with the addition of Tween 80^53^. Our results, however, indicated the opposite trend: the proportions of saturated and mono-unsaturated fatty acids were generally declined with increasing concentration of C9-biosurfactant, whereas the proportion of unsaturated fatty acids tended to increase with increasing C9-biosurfactant concentration mainly due to substantial increase in the proportion of polyunsaturated fatty acids (Tables 2 and 3). The increase in the relative proportion of unsaturated fatty acids has been acknowledged as a compensating response to the decreased thylakoid membrane fluidity under low temperature conditions for a green alga, *Tetraselmis* sp., and an increase in the level of polyunsaturated fatty acids particularly was suggested as a shift in the structure of membrane-forming lipids to spatially increase membrane fluidity that potentially enables more efficient exchange of metabolites^54–56^. Although few studies addressed the influence of biosurfactant on algal membrane fluidity, more careful evaluation on the mechanisms involved in possible shifts in membrane fluidity will be necessary to clearly understand algal response to the additive biosurfactant.

It should be, however, noted that algal response to biosurfactant is closely dependent on the type of biosurfactant and the algal strains that are being cultivated^46,57^. Rieß and Grimme, for instance, reported that diethyleneglycol monohexadecylether (C16E2), a nonionic surfactant, was shown to act as an instantaneous inhibitor of cell growth and reproduction of *Chlorella fusca*, whereas a cationic surfactant cetyltrimethylammonium chloride (CTAC) was shown to induce only minor acute effects on photosynthesis and respiration^57^. A recent study further tested the toxicity of alkylglucosides on the freshwater alga, *Selenastrum capricornutum* and reported that these non-ionic surfactants are relatively innocuous to *Selenastrum* after a 72-hour algal growth inhibition test, while substantial toxicity of these surfactants were confirmed with cladoceran *Daphnia magna*^46^. In addition, Ahn *et al*. confirmed that the additive surfactin slightly inhibited the growth of *Chlorella vulgaris* and a *Scenedesmus* species, suggesting the possibility that two algal strains tested in this study had intrinsically greater tolerance against high surface activity of surfactin than other algal species^58^. These authors, however, also noted that the growth of *Navicula* sp. seemed to be stimulated with the additive surfactin^58^. Therefore, the influence of C9-biosurfactant on a wide variety of algal crop species should be systematically evaluated throughout a long-term operation to successfully achieve the industrial application of C9-biosurfactant as an effective biocontrol agent.

### Applying C9-biosurfactant in industrial algal cultivation platforms

While the results presented in Fig. 1 were based on the cases in which algal cells were not provided throughout the experimental period, it is true that prey availability is not limiting for the growth of algal grazers in industrial algal cultivation platforms^59^. It is thus vitally important to evaluate the effectiveness of C9-biosurfactant in controlling cladoceran grazers in the presence of algal prey because the nutritional status of cladocerans can substantially influence the cladoceran-control activity of C9-biosurfactant^49,50^.

**Figure 5:**
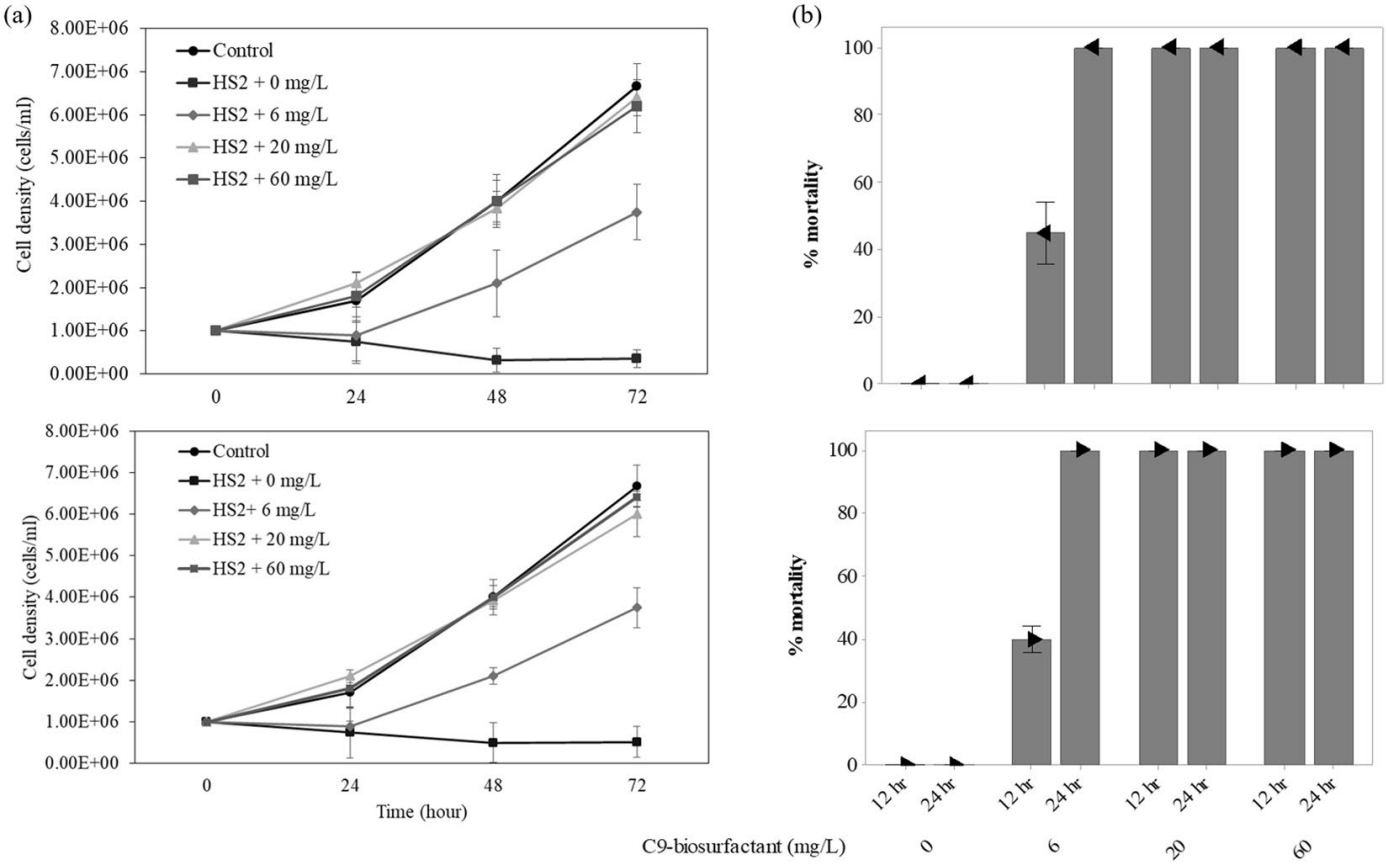
The culture densities of *Chlorella* sp. HS2 under the grazing pressure of *Daphnia pulex* (top) and *Moina macrocopa* (bottom) with varying concentrations of C9-biosurfactant throughout a 72-hour-long cultivation period (**a**) and the corresponding percent mortalities of *Daphnia pulex* (top) and *Moina macrocopa* (bottom) (**b**). Note that no mortalities of both cladocerans were observed in the cultures received 0 mg/L of C9-biosurfactant throughout the experimental period. Error bars represent the standard error of the mean.

**Figure 6:**
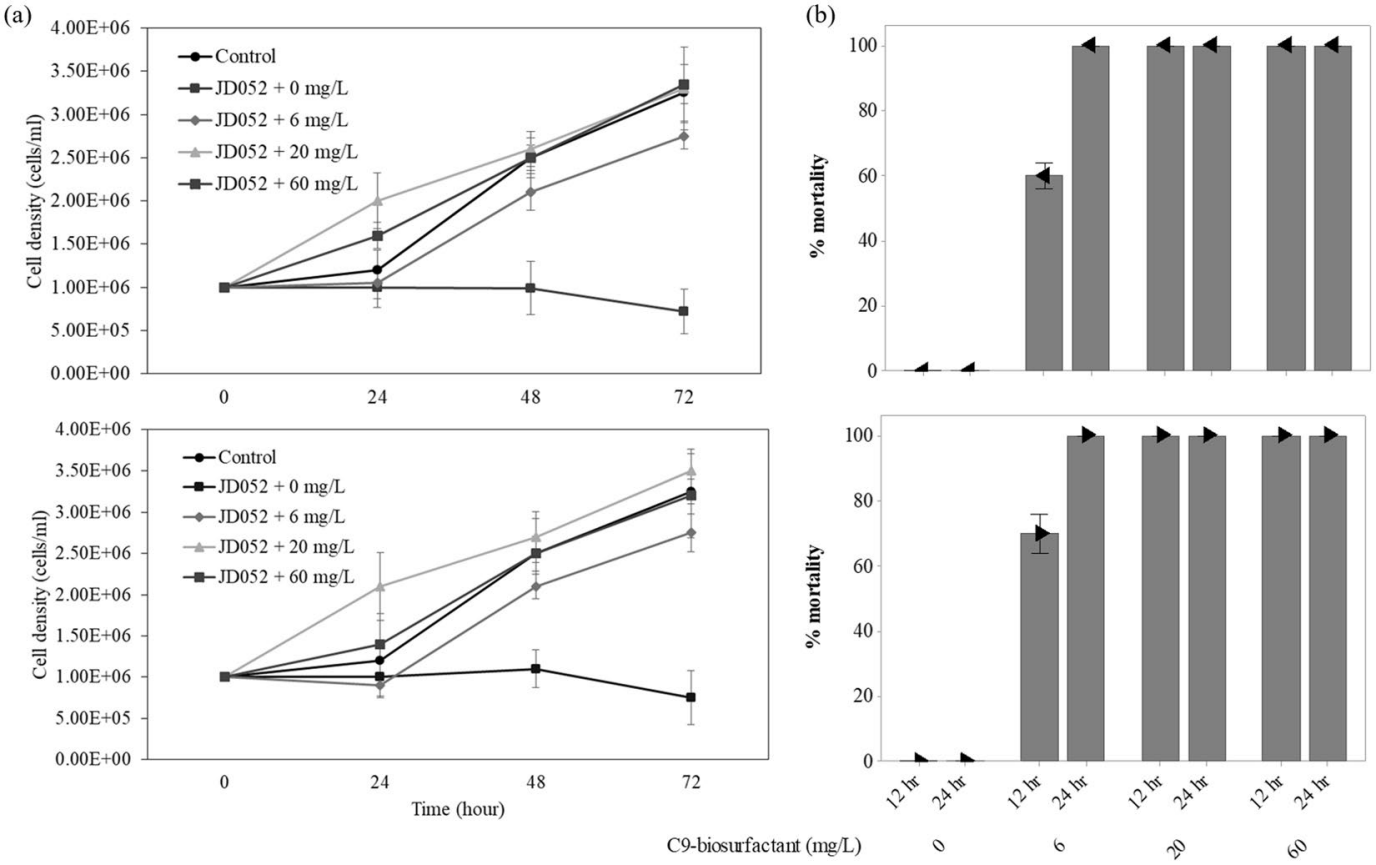
The culture densities of *Scenedesmus deserticola* JD052 under the grazing pressure of *Daphnia pulex* (top) and *Moina macrocopa* (bottom) with varying concentrations of C9-biosurfactant throughout a 96-hour-long cultivation period (**a**) and the corresponding percent mortalities of *Daphnia pulex* (top) and *Moina macrocopa* (bottom) (**b**). Note that no mortalities of both cladocerans were observed in the cultures received 0 mg/L of C9-biosurfactant throughout the experimental period. Error bars represent standard error of the mean.

Although Fig. 1 indicated the complete eradication of cladocerans within 12 hours at the C9-biosurfactant concentration of 6 mg/L, the results clearly showed that more than 12 hours were required to completely eradicate cladoceran grazers with 6 mg/L of C9-biosurfactant under the presence of algal prey (Figs 5b and 6b). In particular, the respective percent mortalities of *Daphnia pulex* were 45 and 60% in the cultures of *Chlorella* sp. HS2 and *Scenedesmus deserticola* JD052, and 40 and 70% of the respective percent mortalities of *Moina macrocopa* were observed in the cultures of *Chlorella* sp. HS2 and *Scenedesmus deserticola* JD052 after treating with 6 mg/L of C9-biosurfactant for 12 hours (Figs 5b and 6b). The delayed mortality response of cladoceran grazers at the C9-biosurfactant concentration of 6 mg/L was reflected in lower algal cell densities than those of the tube cultures amended with higher C9-biosurfactant concentrations (Figs 5a and 6a), and no algal growth limiting effect of C9-biosurfactant was observed at the C9-biosurfactant concentrations above 6 mg/L (Figs 5a and 6a). These results indicate that longer exposure of C9-biosurfactant to cladoceran grazers is likely to be necessary to achieve complete eradication in the presence of algal prey, and reaffirm that the additive C9-biosurfactant does not substantially limit the growth of two selected algal strains.

Although the complete eradication of cladoceran grazers was observed within 12 hours upon the application of C9-biosurfactant at the concentrations of 20 and 60 mg/L, the percent mortalities of cladoceran grazers were greater in the cultures of *Scenedesmus* after 12 hours under 6 mg/L of C9-biosurfactant than in the cultures of *Chlorella*. Such differences in cladoceran mortality may be the results of different ingestion rate of algal grazers by the size of algal prey^14^. Cho *et al*., for example, tested the ingestion rate of *Daphnia* sp. with different algal strains and reported that the large-sized microalga, *Pediastrum* sp. was most resistant to the grazing pressure of *Daphnia*, while relatively smaller unicellular algal species such as *Parachlorella* sp. were effectively grazed by the cladoceran grazer^14^. Cell size and long axis length of microalgae were particularly hypothesized as master traits that inherently control other ecological traits related to the trends in competition and facilitation, especially because size confers a defense against grazing^30,60–62^. Indeed, the cell density of *Chlorella* sp. HS2 was more drastically decreased than that of *Scenedesmus deserticola* JD052 under the presence of cladoceran grazers without supplemental C9-biosurfactant (Figs 5a and 6a). It is therefore likely that small-sized unicellular *Chlorella* sp. HS2 was more effectively ingested by cladoceran grazers than colonial *Scenedesmus deserticola* JD052, and potential differences in algal ingestion efficiency seemed to contribute to the lower percent mortalities of cladoceran grazers in the culture of *Chlorella* sp. HS2 under 6 mg/L of C9-biosurfactant. These results thus reemphasize potential differences in resistance against grazing between different algal species, and further suggest the importance of carefully selecting algal crop species to effectively achieve crop protection against grazing consumers because the extent of crop loss to algal grazers may significantly vary with the choice of algal crop species^30^.

Although the biosurfactants of *Bacillus subtilis* are known to withstand a wide range of pH values, exposure to long hours of sunlight, UV radiation, as well as moist heat up to 121 °C^36,45^, continuous or semi-continuous operation of algal reactors may necessitate greater inputs of C9-biosurfactant to successfully control algal grazers due to typically greater biomass-specific grazing rates than biomass-specific growth rates in continuous cultures^59,63^. In addition, the exposure regime of biocontrol agent (i.e., continuous vs. pulse exposure) was known to play a critical role in determining the effectiveness of selected control agents. While the complete eradication of cladoceran grazers may not be desirable because of the potential ecological imbalances and the establishment of possibly unmanageable alien-grazers^27,30^, future research will need to test the cladoceran-control activity of C9-biosurfactant under varying operational conditions with high-frequency monitoring of ecological communities to assure the industrial applicability of C9-biosurfactant^64^. Furthermore, the environmental impact of the possible release of biosurfactant to waterbodies should be evaluated across different trophic levels to ensure safe and reliable application of C9-biosurfactant, although biosurfactants are generally considered to be less toxic and more biodegradable than synthetic surfactants^34^.

It has to be also noted that the yield of biosurfactant from the culture of *B. subtilis* C9 can be substantially enhanced by optimizing growth medium or selecting cost-effective growth substrate^35,41,65^. Kim *et al*., for example, carefully optimized both macro- and micro- nutrients of the growth medium of *B. subtilis* C9 and achieved the production of C9-biosurfactant at the concentration of 7.0 g/L^35^. Gudina *et al*. further reported 1.31 g/L of average biosurfactant production from *B. subtilis* #573 grown in corn steep liquor (CSL) with the low surface tension values between 29.7 and 31.0 mN/m of cell-free supernatants without dilution^41^. Moreover, Fox and Bala tested waste potato substrates as a low-cost carbon source for surfactant production by *B. subtilis* ATCC 2133, and observed a significant drop of surface tension with the recovered biosurfactant^65^. High yield of biosurfactant from *B. subtilis* with agro-waste particularly indicates the possibility of achieving the production of C9-biosurfactant in a cost-effective manner^41^; further investigation into producing C9-biosurfactant with waste nutrient sources could substantially contribute to the industrial application of C9-biosurfactant as an effective biocontrol agent.

Although there is unlikely to be a one-size-fits-all solution for managing unpredictable blooms of algal grazers at industrial algal biomass production, the results of this study clearly supported the potential of C9-biosurfactant as an effective biocontrol agent for cladoceran algal grazers. The additive C9-biosurfactant, however, did not inhibit the growth and lipid productivity of *Chlorella* sp. HS2 and *Scenedesmus deserticola* JD052, supporting the non-toxicity of C9-biosurfactant to the selected algal crops. While managing algal pests in industrial algal cultivation platforms is likely to require the harmonious utilization of carefully screened control strategies^15^, combining the C9-biosurfactant with other effective biocontrol measures will be needed to successfully reduce or completely eliminate algal grazers. Additionally, we urge more careful investigations into optimizing the application strategy of C9-biosurfactant under varying outdoor cultivation conditions at the scales more relevant to industrial algal cultivation.

## Methods

### Strain selection

#### Bacterial strain

Kim *et al*. previously isolated *Bacillus subtilis* C9 that produces an effective biosurfactant by the method of oil-film collapsing assay^34,35,66^. The biosurfactant of *B. subtilis* C9 was determined to be a lipopeptide consisting of a C_14–15_ fatty acid tail linked to a peptide moiety consisting of 7 amino acid residues that was identical to the peptide moiety of surfactin^34,35,67^. While the lipopeptides produced by *Bacillus* strains are considered as the main compounds involved in the biocontrol activities of *Bacillus* on phytopathogens^31,68^, surfactin is particularly known for its remarkable surfactant properties and interactions with artificial and biomembrane systems (e.g., bacterial protoplasts or enveloped viruses) that were postulated to give rise to its antibiotic activities^34,35,42,48^.

#### Cladoceran algal grazers

While the zooplankton communities of algal cultivation systems are likely to substantially vary across different sites and seasons^39^, *Daphnia pulex* and *Moina macrocopa* were selected as model cladoceran grazers based on their blooms during our year-long operation of wastewater-fed open algal ponds^14^. Laboratory clones of *Daphnia pulex* and *Moina macrocopa* were obtained from a local supplier (Biozoa Biological Supply Co. Ltd, Korea) and Gyeongsang National University (Tongyeong, Korea), respectively. Cladoceran cultures were then maintained at 25 °C in 500-mL beakers at the light intensity of 150 µmol·m^−2^·s^−1^ and 14: 10 L/D photoperiod in COMBO medium^69^. Each zooplankton culture was fed with either *Chlorella* sp. HS2 or *Scenedesmus deserticola* JD052 at the algal cell density of 1 × 10^6^ cells/mL. Cladoceran density at each culture was maintained above 300 individuals/L and each beaker was gently shaken twice a day to ensure that algal cells stayed in suspension.

#### Microalgal strains

While our year-long operation of wastewater-fed open ponds indicated the year-round presence of *Chlorella* and *Scenedesmus* at greater dominance levels than most other algal colonizers^14,70^, *Chlorella* sp. HS2 and *Scenedesmus deserticola* JD052 were previously isolated from local wastewater sources. The bacteria-free seed culture of each of these algal strains was subsequently obtained by treating xenic algal cultures in BG-11 medium with ultrasonication and micropicking^71^. Because the species of *Chlorella* and *Scenedesmus* are particularly recognized for their industrial potential for the production of biofuels and bioproducts^7,8^, we selected these isolated strains to evaluate the influence of C9-biosurfactant on the growth and productivity of algal crops. Prior to the inoculation, samples withdrawn from bacteria-free seed cultures of two algal strains were streaked onto five different types of agar plates that contained organic carbon sources (i.e., YM, R2A, LB, TSA, and BG-11+ 100 ppm of glucose) to assure the absence of culturable bacteria^8,70,71^.

### Production and recovery of biosurfactant from Bacillus subtilis C9

The stock culture of *B. subtilis* C9 was stored at −80 °C in Luria-Bertani (LB) medium supplemented with 20% (v/v) of glycerol. Upon streaking the stock culture on LB agar plates, an inoculum was prepared by inoculating a single colony of *B. subtilis* C9 into 10-mL of LB broth in 125-mL Erlenmeyer flask that was subsequently cultivated at 37 °C for 12 hours. The 12-hour-old inoculum was used to seed 200-mL of LB broth medium supplemented with 5 g/L of glucose in each of triplicated 1-L culture flasks; the initial optical density of each culture following inoculation was 0.05 at 600 nm and three culture flasks were incubated at 37 °C for 72 hours on an orbital shaker at 170 rpm. The composition of LB medium was (g/L): NaCl 10.0; tryptone 10.0; yeast extract 5.0^41,72^.

The recovery of C9-biosurfactant was performed as described in previous studies^34,35,41^. Briefly, the cultures were first centrifuged at 7500 rpm for 20 minutes to remove bacterial cells after a 72-hour-long cultivation period. Upon confirming the production of biosurfactant by measuring the surface tension of culture broth with a ring tension meter at 25 °C (K10ST; Kruss, Hambrug, Germany), the cell-free supernatants were adjusted to pH 2 with 6 M HCl and were subsequently incubated overnight at 4 °C to promote the precipitation of biosurfactant^34,35,41^. The precipitates were then collected by centrifugation at 10000 rpm for 20 minutes at 4 °C. Afterwards, the biosurfactant of *B. subtilis* C9 was dissolved in a minimal amount of demineralized water and biosurfactant solution was subsequently lyophilized after adjusting pH to 7 using 1 M NaOH^41^. The freeze-dried products were weighed to calculate the yield of biosurfactant prior to storing at −20 °C. A known volume of culture medium was used to dissolve the recovered biosurfactant of *B. subtilis* C9 to explore its biocontrol activity against cladoceran algal grazers and its influence on the growth and productivity of selected algal strains under different concentration gradients. Although direct quantification of surfactin in C9-biosurfactant was not performed in this study, the relative proportion of surfactin in C9-biosurfactant was estimated by comparing critical micelle dilution (CMD) of aqueous solutions of C9-biosurfactant and HPLC-grade surfactin at an identical concentration of 0.3 g/L. CMD was the dilution necessary to reach the point where the surface tension starts to dramatically increase, and was known to be proportional to the amount of surfactin present in the original sample^34,73^.

### Experimental design

#### Assessment of the biocontrol activity of C9-biosurfactant against cladoceran algal grazers

*Chlorella-*fed adult cladocerans were first gently harvested by filtering the liquid with a 500 µm mesh filter and carefully rinsed with clean COMBO medium to remove any debris. Subsequently, each of 10-mL glass tubes received 10 individuals of either *Daphnia pulex* or *Moina macrocopa*. The average adult body sizes of *Daphnia pulex* and *Moina macrocopa* were 1.8 ± 0.3 mm and 1.2 ± 0.2 mm, respectively. Each glass tube was then tested at C9-biosurfactant concentrations of 0, 6, 20, and 60 mg/L in plain COMBO medium. Alive and dead individuals were counted thrice after 1, 12, and 24 hr by considering the cladocerans settled onto the bottom of glass tube without visual movement after gentle shaking as dead. In addition to the recovered C9-biosurfactant, HPLC-grade surfactin from *B. subtilis* (Sigma-Aldrich, St. Louis, MO, USA) was tested under identical experimental conditions at four concentrations (0, 6, 20, and 60 mg/L) in plain COMBO medium to confirm whether the main surfactant compound of C9-biosurfactant is primarily contributing to its cladoceran control activity^34,35^. All experiments were performed in a temperature-controlled room at 25 ± 1 °C under the light intensity of 150 µmol·m^−2^·s^−1^, and the pH level of each tube culture ranged between 7.0 and 8.0 throughout the experimental period, ensuring no significant influence of ammonia toxicity on the mortality of algal grazers^74^.

#### Influence of C9-biosurfactant on the growth of selected algal crops

The influence of C9-biosurfactant on the growth of *Chlorella* sp. HS2 and *Scenedesmus deserticola* JD052 was tested to determine whether C9-biosurfactant negatively influenced the growth and productivity of selected algal strains. Prior to inoculation, triplicated 250-mL Erlenmyer flasks were subjected to four different concentrations of C9-biosurfactant (0, 6, 20, and 60 mg/L) in plain BG-11 medium. Either *Chlorella* sp. HS2 or *Scenedesmus deserticola* JD052 was then inoculated into each flask culture at the cell density of 1 × 10^6^ cells/mL with the final working volume of 100-mL. Each flask culture was supplemented with 5% CO_2_ through an autoclavable aeration tube with an inner diameter of 1 mm at 0.1 volume gas per volume culture per minute (vvm), and all cultures were shaken at 100 rpm on an orbital shaker under continuous light at the intensity of 150 µmol·m^−2^·s^−1^. Flask cultures were harvested two days after stationary growth phase was reached, and the harvested biomass was subsequently lyophilized to explore the influence of C9-biosurfactant on the biomass and lipid productivity of two algal strains. In addition, *Chlorella* sp. HS2 and *Scenedesmus deserticola* JD52 were cultured in 24-well plates without supplemental CO_2_ at four different concentrations of HPLC-grade surfactin from *B. subtilis* (0, 6, 20, and 60 mg/L) in plain BG-11 medium to confirm whether the main surfactant compound of C9-biosurfactant negatively influenced the growth of selected algal species under continuous light at the intensity of 150 µmol·m^−2^·s^−1^. The final working volume for each of quintuplicated wells was 1.5 mL and the well plates were shaken at 100 rpm on an orbital shaker after covering with PARAFILM^®^ to minimize evaporative loss. Both flask and well plate experiments were performed in a temperature-controlled room at 25 ± 1 °C.

#### Exploring the biocontrol activity of C9-biosurfactant in grazer-introduced algal cultures

Because blooms of cladoceran grazers in algal cultivation systems will occur under the presence of algal crops, the grazer-control activity of C9-biosurfactant was further evaluated by monitoring the percent mortality of cladoceran grazer in algal cultures at different concentrations of C9-biosurfactant. Each of 10-mL glass tubes was first inoculated with either *Chlorella* sp. HS2 or *Scenedesmus deserticola* JD052 at the cell density of 1 × 10^6^ cells/mL, and subsequently received 10 individuals of either *Daphnia pulex* or *Moina macrocopa* that were carefully rinsed with clean COMBO medium after being fed for at least 48 hours with the identical algal strain of the culture they were added. Each of triplicated tube cultures was then subjected to four different concentrations of C9-biosurfactant (0, 6, 20, and 60 mg/L) in plain COMBO medium at 25 ± 1 °C under the light intensity of 150 µmol·m^−2^·s^−1^. Both alive and dead cladocerans were counted every 12 hours, and algal cell density in each culture was monitored daily throughout a 72-hour-long experimental period. In addition, the growth of each algal strain without the presence of cladoceran grazers was monitored for 72 hours in triplicated tube cultures in plain COMBO medium under identical light and temperature conditions. Because dissolved CO_2_ and agitation may negatively influence the survival of cladocerans by limiting the exchange of CO_2_ and exerting shear stress, all tube cultures were only gently shaken twice a day without supplemental CO_2_ to ensure that algal cells stayed in suspension^27,43^.

### Analytical methods

#### Identification of C9-biosurfactant by mass spectrometry

While previous studies indicated a lipopeptide surfactin as the main surfactant compound of C9-biosufactant, the recovered C9-biosurfactant and HPLC-grade surfactin from *Bacillus subtilis* (Sigma-Aldrich, St. Louis, MO, USA) were submitted to mass spectrometry analysis after dissolving each compound into methanol at the concentration of 1 mg/L^34,35^. High-resolution mass spectra were collected using a Bruker micrOTOF-Q II mass spectrometer (Bruker Daltonics, Bremen, Germany) in ESI positive ion mode over the mass range of *m/z* 50–2000 under the following optimized settings: end plate offset, −500 V; capillary, 4800 V; nebulizer gas, 0.4 bar; dry gas, 4.0 liters/min; dry gas temperature, 180 °C. The obtained spectra of C9-biosurfactant and HPLC-grade surfactin were subsequently compared to assure the presence of surfactin in the recovered C9-biosurfactant.

#### Algal growth measurement

Culture growth was measured by either counting cell numbers (i.e., microalgal cell density) under a Nikon FX-A microscope (Optical Analysis Corp., Nashua, Japan) with a hemocytometer (C-chip, NanoEnTek, USA) or measuring optical density at 680 nm using a microplate reader (BioTek, Bad Friedrichshall, Germany). The dry cell weight (DCW) was also determined by filtration onto pre-weighed GF/C filters (Whatman, UK). After rinsing with distilled water, the filters were dried at 105 °C overnight. The filters were then reweighed after drying and the DCW was calculated from the difference between the filter weight with and without algal biomass. In addition, the specific growth rate of each flask culture was calculated during exponential growth phase as:

1 $$\mu =ln(\frac{{C}_{2}}{{C}_{1}})\times \frac{\,1}{{t}_{2}-{t}_{1}}$$

where µ was the specific growth rate, and C_1_ and C_2_ were culture densities at time 1 (t_1_) and time 2 (t_2_), respectively.

#### Lipid content and fatty acid methyl ester (FAME) composition of harvested algal biomass

Each of 250-mL flask cultures was harvested at the end of batch cultivation period by removing supernatant after centrifugation at 4000 rpm for 10 min, and the harvested biomass was subsequently lyophilized and stored in the freezer at −70 °C until further analysis. The lipid content of harvested biomass was analyzed by extracting total lipids from freeze-dried biomass with chloroform-methanol (2:1 (v/v)) following a slightly modified version of Bligh and Dyer’s method^7,75,76^. FAME composition of lyophilized biomass was analyzed using a gas chromatograph (Shimadzu GC-2010, Japan). Each of 50 mg samples was first placed into capped test tubes, saponified with 1 mL of a saturated KOH-CH_3_OH solution at 75 °C for 10 min, and submitted to methanolysis with 5% HCl in methanol at 75 °C for another 10 min^8,77^. Fatty acids containing phase was subsequently separated by adding 2 mL of distilled water and recovered^8,77^. The components were identified by comparing their retention times and peak areas with those of FAME Mix, C8-C24 (18918-1AMP, Supelco, Sigma-Aldrich, St. Louis, MO, USA)^8,76,77^.

#### Algal gazer mortality

The % mortality of cladoceran grazer in each tube culture was calculated as:

2 $$ \% \,{\rm{mortality}}=(\frac{{n}_{0}-{n}_{t}}{{n}_{0}})\times 100$$

where n_0_ was the total number of cladoceran grazer at time 0, and n_t_ was the number of alive individuals at each time point. The individuals settled onto the bottom of tube culture without visual movement after gentle shaking were considered as dead, and no newborn cladocerans were observed across all experimental treatments.

#### Statistical analysis

The results were presented as either mean values or mean values ± standard deviation from at least triplicated independent measurements unless denoted otherwise. The statistical significance of differences in specific growth rate and lipid productivity between treatments was evaluated by using 95% confidence intervals for the mean.

### Data availability

The datasets generated during and/or analyzed during the current study are available from the corresponding authors on reasonable request.

## Electronic supplementary material

Supplementary Information
