# Author Correction: Application of biosurfactant from *Bacillus subtilis* C9 for controlling cladoceran grazers in algal cultivation systems

**DOI:** 10.1038/s41598-020-60509-1

**Published:** 2020-02-20

**Authors:** Jin-Ho Yun, Dae-Hyun Cho, Bongsoo Lee, Hee-Sik Kim, Yong Keun Chang

**Affiliations:** 1 Department of Chemical and Biomolecular Engineering, KAIST, 291 Daehak-ro, Yuseong-gu, Daejeon 305-701 Republic of Korea; 2 Cell Factory Research Center, Korea Research Institute of Bioscience and Biotechnology (KRIBB), Yuseong-gu, Daejeon 305-806 Republic of Korea; 3 Green Chemistry and Environmental Biotechnology, University of Science and Technology (UST), Yuseong-gu, Daejeon 305-350 Republic of Korea; 4 Advanced Biomass R&D Center, 291 Daehak-ro, Yuseong-gu, Daejeon 305-701 Republic of Korea

Correction to: *Scientific Reports* 10.1038/s41598-018-23535-8, published online 29 March 2018

This Article contains an error in the Methods section in the ‘Strain selection’ subsection under the subheading ‘Microalgal strains’,

“While our year-long operation of wastewater-fed open ponds indicated the year-round presence of *Chlorella* and *Scenedesmus* at greater dominance levels than most other algal colonizers^14,70^, *Chlorella* sp. HS2 and *Scenedesmus deserticola* JD052 were previously isolated from local wastewater sources.”

should read:

“While our year-long operation of wastewater-fed open ponds indicated the year-round presence of *Chlorella* and *Scenedesmus* at greater dominance levels than most other algal colonizers^14,70^, *Chlorella* sp. HS2 and *Scenedesmus deserticola* JD052 were previously isolated from oligo-mesotrophic water bodies.”

