# Supplementary material for: Application of biosurfactant from Bacillus subtilis C9 for controlling cladoceran grazers in algal cultivation systems
Source: Sci Rep. 2018 Mar 29;8:5365. doi: 10.1038/s41598-018-23535-8 (PMC5876376; doi:10.1038/s41598-018-23535-8)

Supplementary Information

Application of biosurfactant from *Bacillus subtilis* C9 for controlling cladoceran grazers in algal cultivation systems

Jin-Ho Yun[a], Dae-Hyun Cho[b], Bongsoo Lee[a], Hee-Sik Kim[b,c,*], Yong Keun Chang[a,d,*]

[a]Department of Chemical and Biomolecular Engineering, KAIST, 291 Daehak-ro, Yuseong-gu, Daejeon 305-701, Republic of Korea

[b]Cell Factory Research Center, Korea Research Institute of Bioscience and Biotechnology (KRIBB), Yuseong-gu, Daejeon 305-806, Republic of Korea

[c]Green Chemistry and Environmental Biotechnology, University of Science and Technology (UST), Yuseong-gu, Daejeon 305-350, Republic of Korea

[d]Advanced Biomass R&D Center, 291 Daehak-ro, Yuseong-gu, Daejeon 305-701, Republic of Korea

[*]Corresponding authors:

Hee-Sik Kim

Tel: +82 42 860 4326; Fax: +82 42 860 4594

E-mail address: hkim@kribb.re.kr

Yong Keun Chang

Tel: +82 42 350 3927; Fax: +82 42 350 3910

E-mail address: ychang@kaist.ac.kr

Fig. S1. High-resolution mass spectra of C9-biosurfactant (top) and HPLC-grade surfactin from *Bacillus subtilis* C9 (Sigma-Aldrich, St. Louis, MO, USA) (bottom). Both spectra exhibited the highest peak at *m/z* 1058.67, where the sodium adduct of surfactin is putatively present.

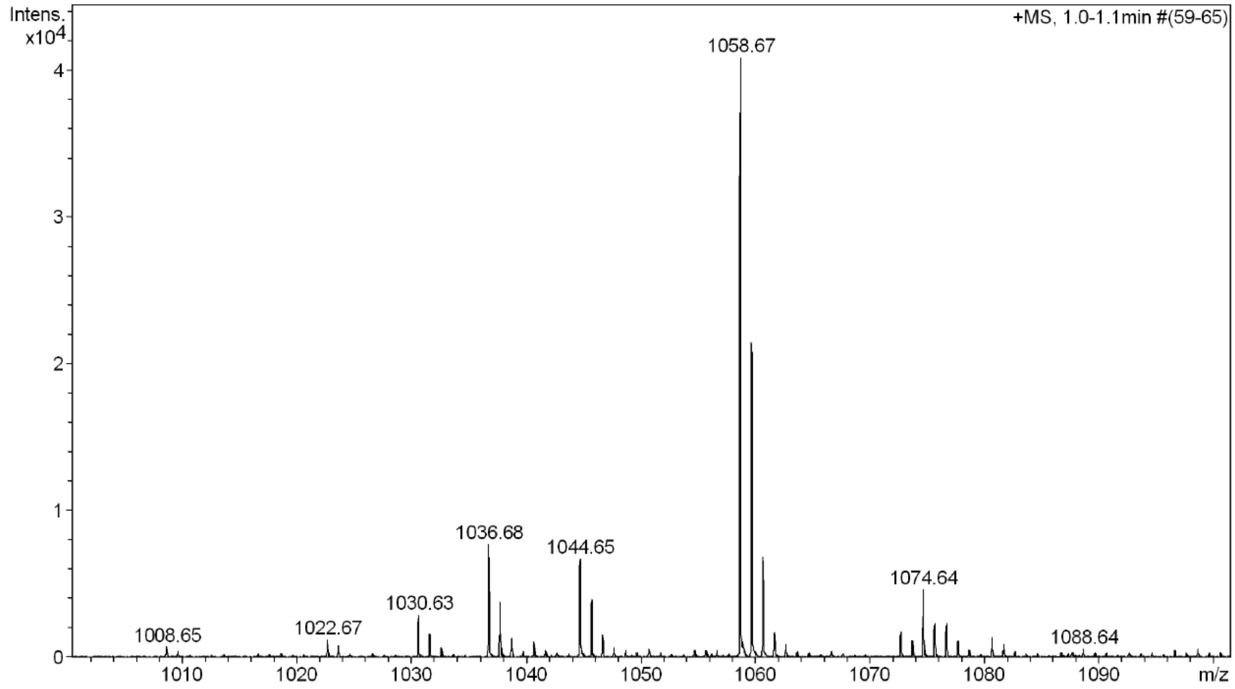

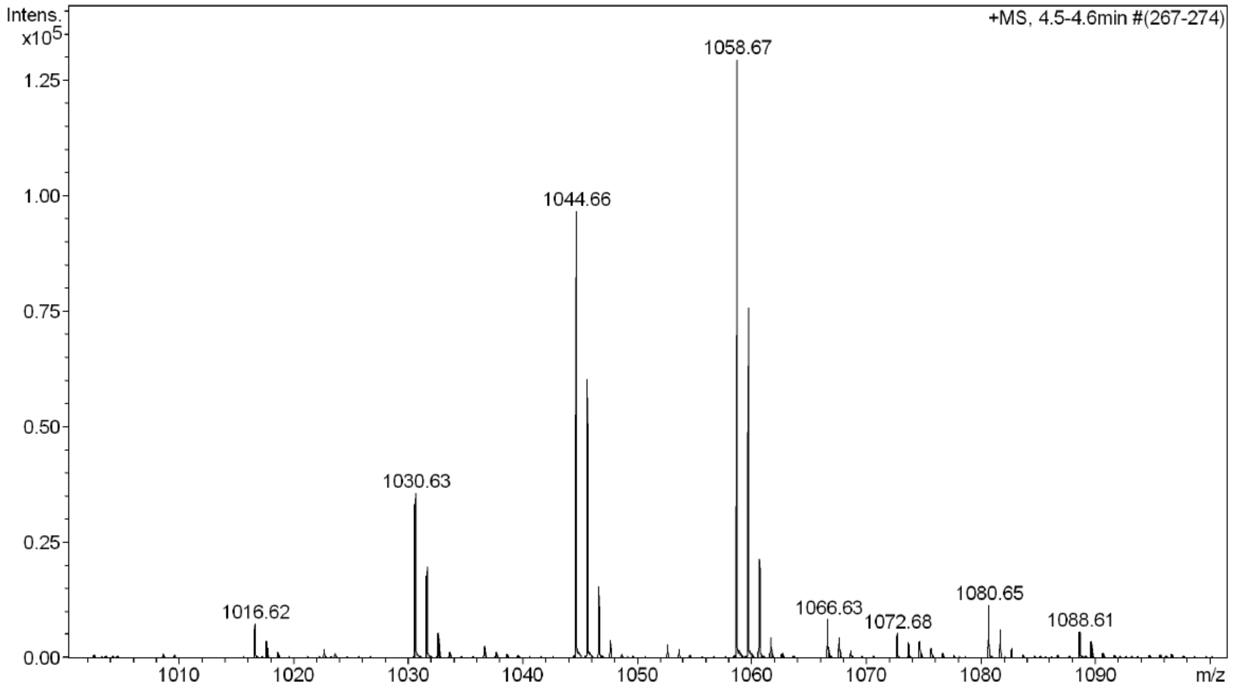

Fig. S2. Growth curve of *Bacillus subtilis* C9 throughout a 72-hour-long cultivation period in 1-L culture flasks under conditions described in the methods section. Bacterial growth was monitored by measuring optimal density at 600 nm, and critical micelle dilution (CMD) of each of culture broth and the aqueous solution of HPLC-grade surfactin at the concentration of 0.3 g/L was also determined as the dilution necessary to reach the point where the surface tension started to dramatically increase. Error bars denote the standard deviation from the mean of triplicate independent measurements.

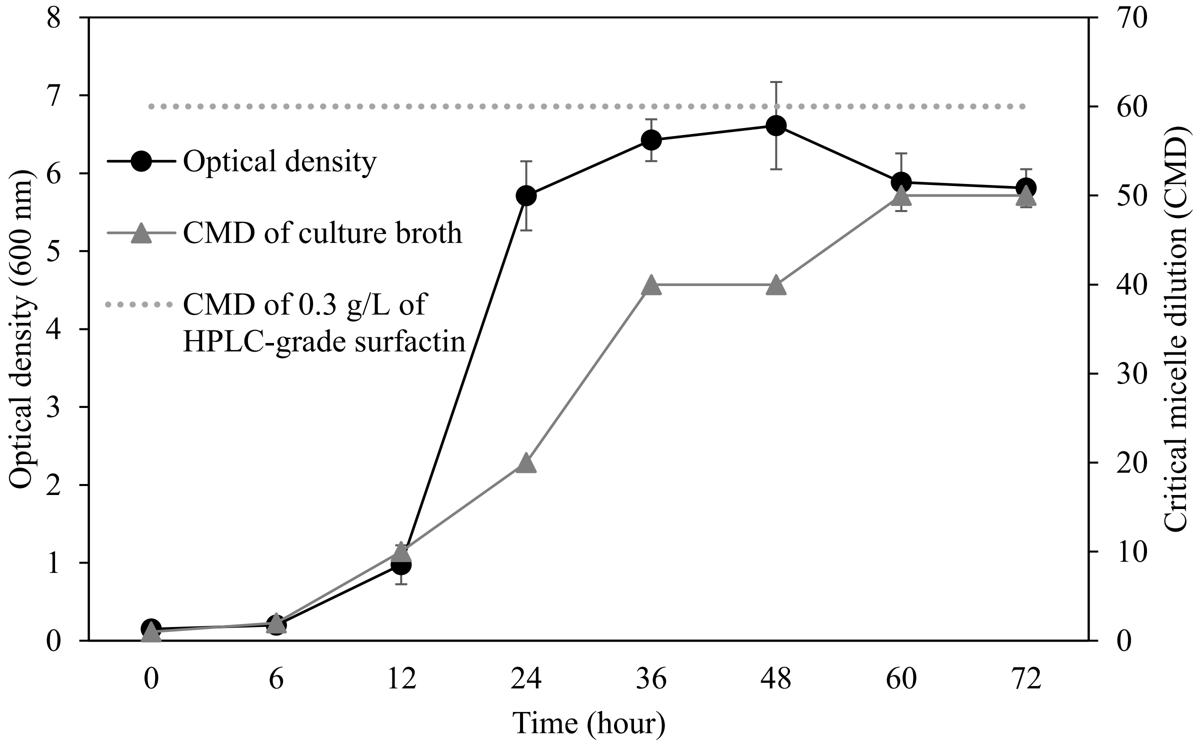

Fig. S3. Standard curve representing the linear relationship between the concentration of HPLC-grade surfactin and critical micelle dilution (CMD). CMD was determined as the dilution necessary to reach the point where the surface tension started to dramatically increase.

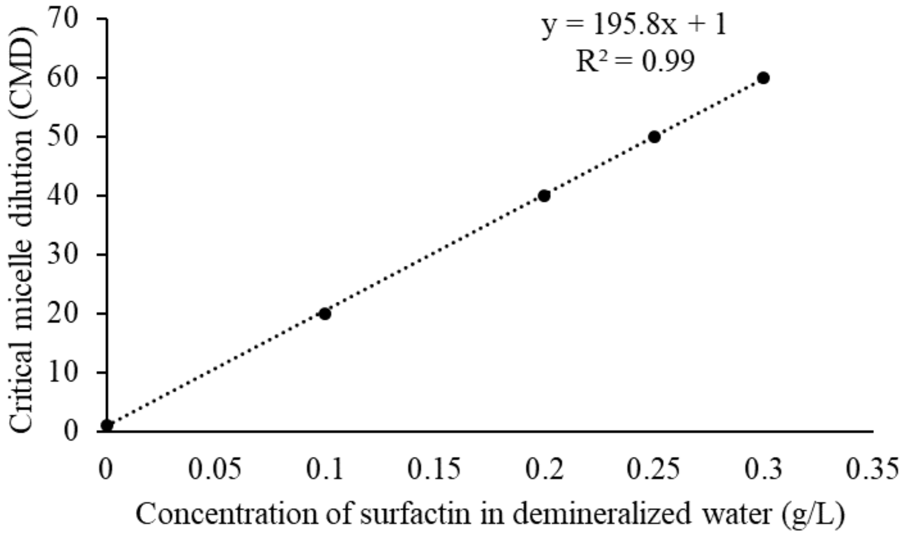

Fig. S4. Photomicrographic images of alive (left) and dead (right) individuals of *Daphnia pulex* (a) and *Moina macrocopa* (b) under varying concentrations of C9-biosurfactant (scale bar denotes 0.5 mm).

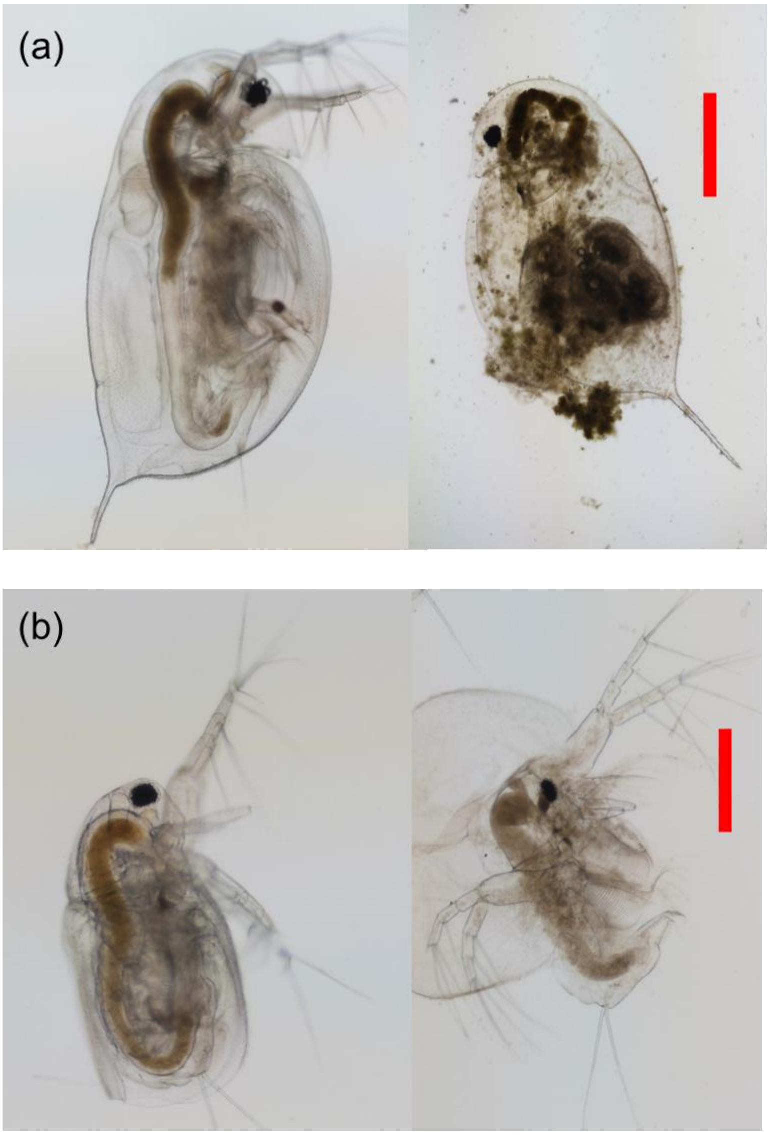

Fig. S5. The % mortalities of *Daphnia pulex* (a) and *Moina macrocopa* (b) after 1, 12, and 24 hr of exposure to HPLC-grade surfactin from *Bacillus subtilis*. Individuals settled on the bottom of 10-mL glass tubes without visual movement after gentle shaking were counted as dead. Error bars denote standard error of the mean.

(a)

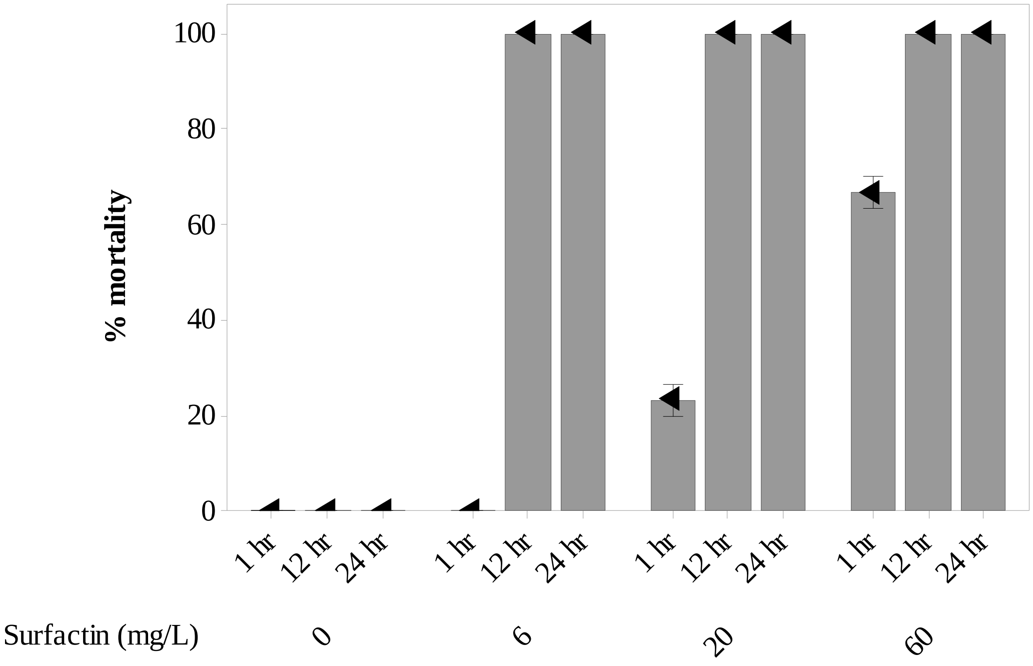

(b)

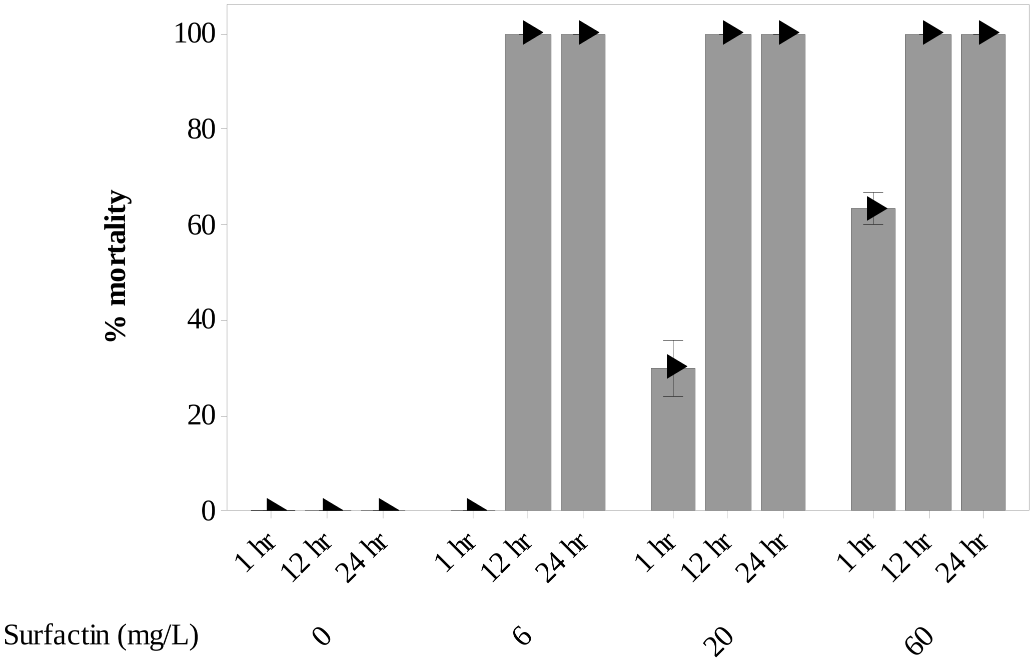

Fig. S6. Photomicrographic images of *Chlorella* sp. HS2 (left) and *Scenedesmus deserticola* JD052 (right) (scale bar denotes 10 μm).

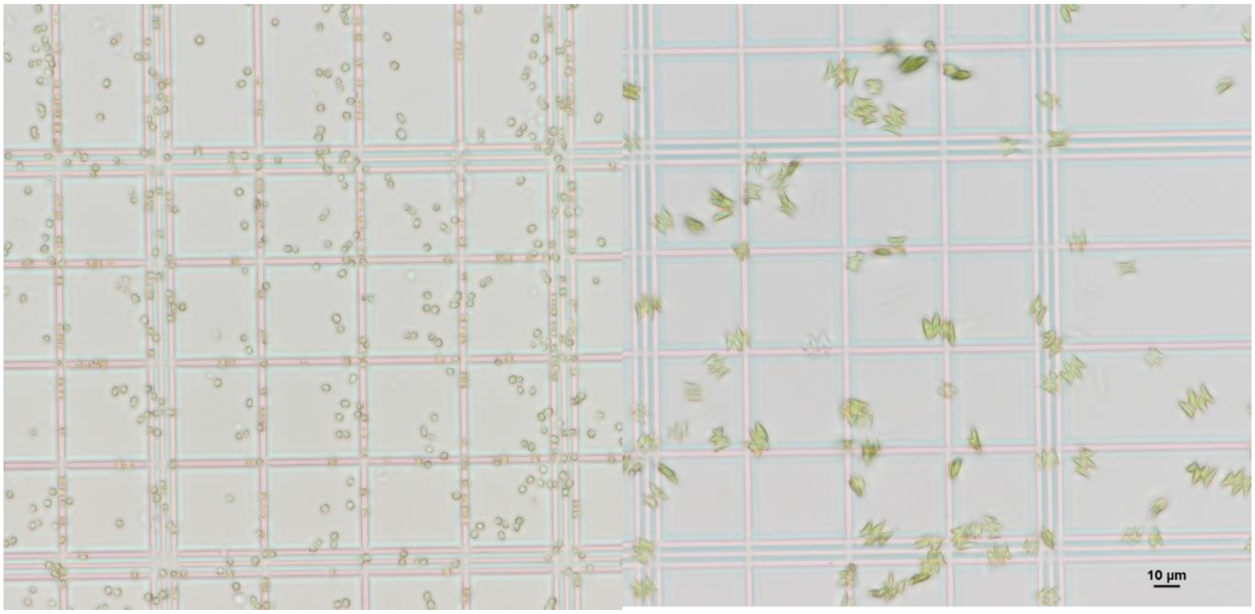

Fig. S7. The growth curves of *Chlorella* sp. HS2 (a) and *Scenedesmus deserticola* JD052 (b) under varying concentrations of HPLC-grade surfactin from *Bacillus subtilis* in 24-well plates. While the OD values were monitored throughout the experimental periods, the corresponding cell density of each well on the final day was used to extrapolate the cell density throughout the entire growth periods. Error bars denote the standard deviation from the mean of quintuplicate independent measurements.

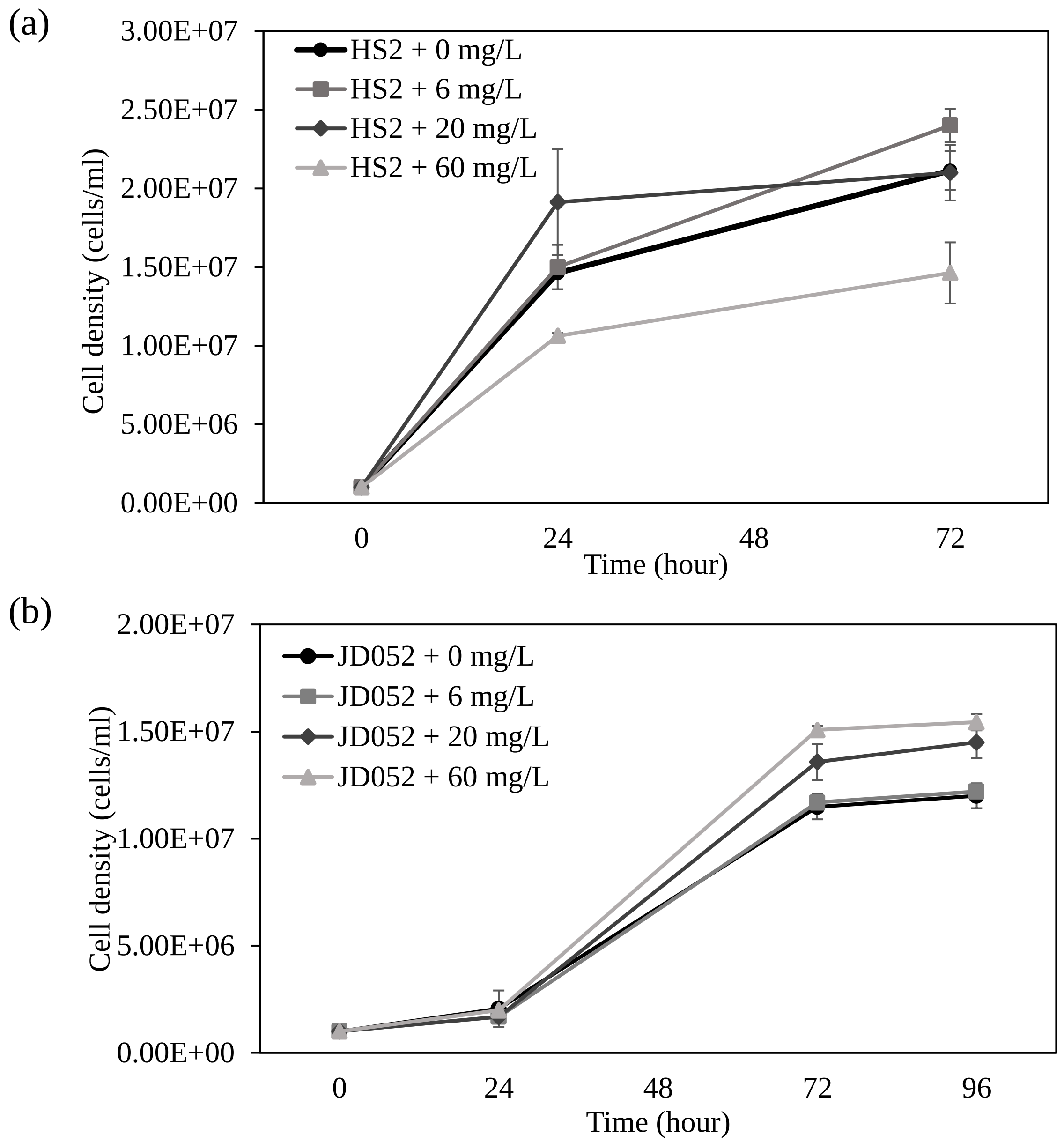

Fig. S8. The percent mortalities of *Daphnia pulex* in differently diluted culture broth of *Bacillus subtilis* C9. *B. subtilis* C9 grown in LB medium for 72 hours was tested for cladoceran control activity under identical conditions as described in the methods section after diluting the culture broth in COMBO medium at the concentrations (v/v %) of 0 (control), 1, 10, and 20. Error bars represent standard error of the mean.

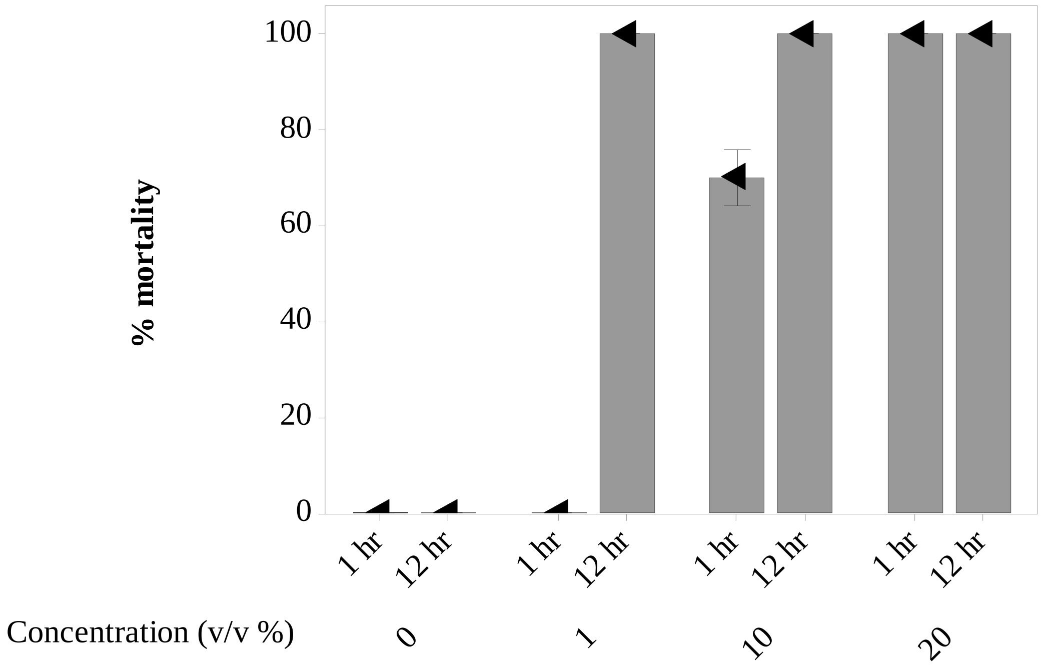

Fig. S9. Growth curves of *Chlorella* sp. HS2 with and without *Bacillus subtilis* C9 in sterile 250-mL flasks at 25 °C under continuous light at the intensity of 170 μmol·m^{-2}·s^{-1} without supplemental CO_2. Each algal culture was initially inoculated with bacteria-free seed culture at the initial cell density of 1 × 10^6 cells/mL. Bacterial cells were then added into corresponding flasks after rinsing the harvested pellets of *B. subtilis* C9 with sterile BG-11 medium. The initial cell density of *B. subtilis* C9 upon inoculation was 1 × 10^6 cells/mL, and the growth curve of *B. subtilis* C9 in the culture of *Chlorella* sp. HS2 was obtained by enumerating bacterial cells throughout the cultivation period under Olympus BX51 fluorescence microscope (Olympus, Japan) after staining subsamples with SYBR-II. Error bars denote the standard deviation from the mean of triplicate independent measurements.

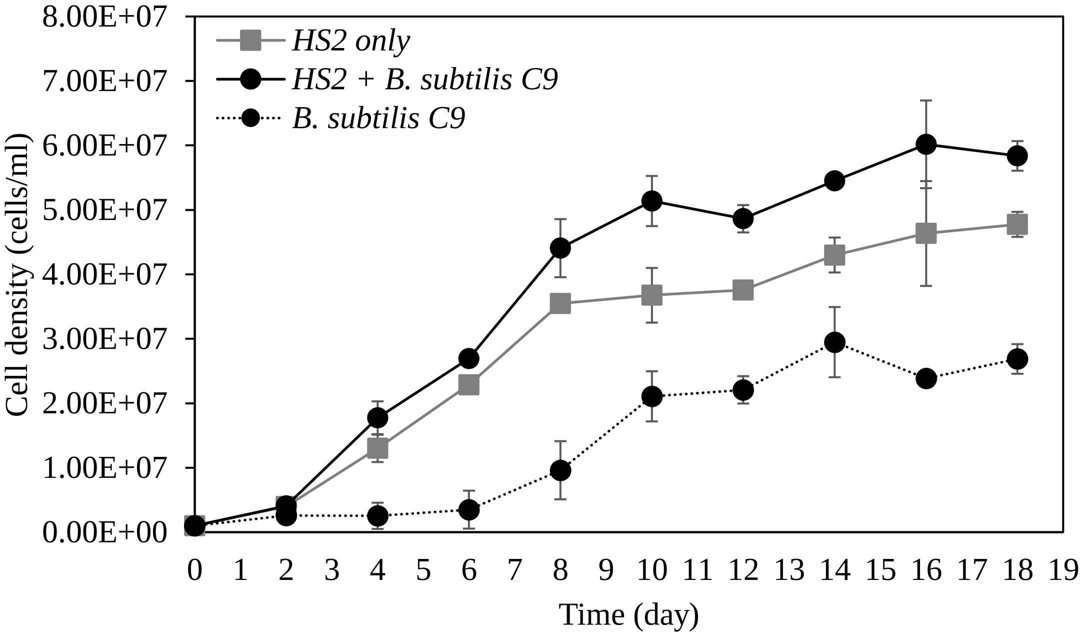

Supplement: Supplementary file 1 — Supplementary Information [file 41598_2018_23535_MOESM1_ESM.pdf]
